# A Rare Case of Statin-induced Immune-mediated Necrotizing Myopathy

**DOI:** 10.7759/cureus.7500

**Published:** 2020-04-01

**Authors:** Anantha Sriharsha Madgula, Naga Vaishnavi Gadela, Meghana Singh, Kai Chen

**Affiliations:** 1 Internal Medicine, University of Connecticut School of Medicine, Farmington, USA; 2 Internal Medicine, University of Connecticut, Farmington, USA; 3 Internal Medicine, University of Connecticut, Hartford, USA; 4 Cardiology, University of Connecticut Health Center, Farmington, USA

**Keywords:** statin-induced necrotizing autoimmune myopathy, drug-related side effects and adverse reactions, coronary artery disease, autoimmune

## Abstract

Statin-associated myopathy comprises of a spectrum of conditions ranging from benign myalgias to statin-induced immune-mediated necrotizing myopathy. Statin-induced immune-mediated necrotizing myopathy is an autoimmune condition wherein there is a destruction of normal skeletal muscular architecture that can be severely debilitating if not recognized promptly. Given its rarity, management is a challenge. We present one such case that was managed with aggressive immunosuppression.

## Introduction

Statins are widely used for primary and secondary prevention of coronary artery disease (CAD). The most commonly reported side effect is proximal muscle myopathy that can range from self-limiting episodes of myalgias to statin-induced immune-mediated necrotizing myopathy (IMNM) [1,2]. More recently, an antibody directed against 3-hydroxy-3-methylglutaryl-coenzyme A reductase (HMGCR) has been identified and is implicated as a culprit among the extreme cases of IMNM [3,4]. We present a case report of a patient with IMNM, who was on statins for CAD.

## Case presentation

A 66-year-old male with a history of CAD presented to our hospital with a chief complaint of generalized weakness. He reported that symptoms began four months before this presentation with generalized muscle aches and weakness, mostly in shoulders and hips. Given his occupation as an entomologist and history of multiple episodes of Lyme disease, he was treated with a three-week course of doxycycline that led to no improvement. One month later, he was admitted to an outside hospital with similar complaints. During that admission, the patient was noted to have a diffuse erythematous rash over upper eyelids and anterior chest wall, and his serum creatine kinase (CK) was elevated to greater than 14,000 units per liter (U/L). He was given a presumed diagnosis of dermatomyositis and was started on treatment with pulse dose intravenous methylprednisolone for three days and then discharged on 60 milligrams of prednisone daily and 15 milligrams of weekly methotrexate, with some improvement in symptoms. His statin was stopped on discharge. The rash gradually improved over the next month, but his muscle weakness progressively worsened to a point where he required assistance with walking and developed difficulty swallowing. He was then admitted to our hospital when physical examination showed a confluent dissipating erythematous rash but significant weakness in all muscle groups. Regarding his past medical history, the patient suffered a myocardial infarction three years earlier, when he was treated with drug-eluting stents in his right coronary artery. Since then he had been on high-intensity atorvastatin 80 milligrams daily for secondary prevention.

**Figure 1:**
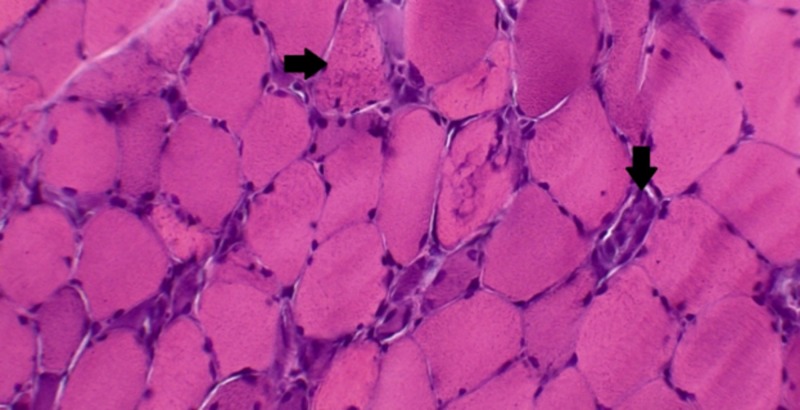
A muscle biopsy sample with hematoxylin and eosin staining A muscle biopsy sample with hematoxylin and eosin staining demonstrating the loss of normal muscle architecture, and extensive myofiber necrosis (right arrow). Macrophages are seen scavenging the necrotic muscle tissue (downward arrow) with sparse regenerating myofibers. Macrophages are the predominant infiltrating cell type, with minimal neutrophilic infiltration.

In our hospital, extensive blood work was done, which showed CK level of 3,669 U/L, erythrocyte sedimentation rate 50 millimeters/hour, aspartate aminotransferase 422 U/L, alanine aminotransferase 669 U/L, and alkaline phosphatase 84 U/L. Workup for malignancy was pursued given its association with dermatomyositis, including a CT scan of chest, abdomen, and pelvis showed no concerning features. Myositis panel was sent, and a muscle biopsy was planned concurrently. His myositis panel came back negative for Jo-1, PL-7, PL-12, Mi-2, Ku, EJ, OJ, and SRP autoantibodies; however, he was tested positive for the anti-HMGCR antibody. His muscle biopsy showed myofiber necrosis and myophagocytosis associated with abundant regenerating myofibers with no evidence of inflammation (Figure 1). Overall, these findings were consistent with a diagnosis of statin-induced IMNM.

Due to progressively worsening muscle weakness, he was started on intravenous methylprednisolone 60 milligrams every six hours, intravenous immunoglobulin (IVIG), and rituximab initially. Because of deteriorating dysphagia, a percutaneous endoscopic gastrostomy (PEG) tube was placed to ensure adequate nutrition. After the diagnosis of IMNM was made, his regimen was switched to cyclophosphamide, IVIG monthly for three months, mycophenolate 1,000 milligrams twice daily, trimethoprim-sulfamethoxazole double strength for pneumocystis prophylaxis, and prednisone taper. Over the course of hospitalization, he developed respiratory failure requiring intubation and eventually tracheostomy. He was then transitioned to an acute rehabilitation facility with a tracheostomy tube and a PEG tube. In response to the treatment, his CK levels trended down to normal range over several weeks. His muscle weakness did not show significant improvement in the hospital.

## Discussion

Statin-induced myopathy has a spectrum of phenotypes that consist of statin-induced myalgia at one end where muscle pain is not associated with elevation of serum CK levels, statin-induced myositis where muscle pain is associated with CK level elevation, statin-induced rhabdomyolysis with marked elevation of CK levels, and IMNM which presents with necrosis without inflammation [1].

IMNM is a rare entity with an estimated incidence of two to three new cases for every 100,000 patients exposed to statins and was first described by researchers from Johns Hopkins Myositis Center in Baltimore. Myofiber necrosis without prominent inflammation was previously a non-specific finding in patients with dystrophies and toxic or immune-mediated myopathies. These patients were treated with immunosuppression with improvement in symptoms. In that study, a novel autoantibody termed "anti-200/100 autoantibody" was found to be associated with proximal weakness in a 100% of the patients with the mean maximum CK level of 10,333 U/L [3]. The same group later identified that statins upregulate the expression of HMGCR, the primary target of autoantibodies in statin-associated IMNM. They also recognized that regenerating muscle cells express high levels of HMGCR, which may implicate that immune response sustains even after discontinuation of statins [4].

Usually, the first presentation is with progressive proximal muscle weakness involving pectoral and pelvic limb girdles. These symptoms do not improve despite the withdrawal of statins, which may help differentiate from other benign statin-induced myopathies [2,5,6]. Testing of serum CK levels should follow, and if alarming levels are detected, patients should be appropriately referred to specialists. In a recent case series study of 12 patients, the mean duration of atorvastatin therapy before first CK elevation in IMNM was 38.8 months, as compared to one month for the onset timing of most statin-induced muscle adverse events. Moreover, there was no statin dose-response effect noted in IMNM [7]. A definitive diagnosis can be made by serology or muscle biopsy. Muscle biopsy usually shows necrosis with the regeneration of muscle fibers and scarce inflammation, mainly composed of macrophages. The serologic diagnosis is by testing for anti-HMGCR antibodies [2,5,6,8].

Immunosuppression remains the cornerstone of therapy. As with our patient, treatment is guided by rheumatology with multidisciplinary coordination. High-dose corticosteroids are given initially, and further immunosuppression can be with methotrexate, azathioprine, or mycophenolate. There is no robust evidence that can differentiate among these medications. Rituximab has also been used in some cases [1,9]. If there is no clinical response with these medications, IVIG can be used and has shown improvement [2,5]. It was suggested that IVIG may attenuate the autoimmune process, allowing muscle regeneration to outpace muscle destruction, but may not wholly abolish muscle degeneration [5]. More recently, Meyer et al. showed that in selective cases, initial induction with triple steroid/IVIG/steroid-sparing agent may be more efficacious than induction with steroids alone [10].

Statins have become an indispensable part of therapy for CAD. However, re-challenging with a statin in patients with IMNM is unlikely a wise option. Other cholesterol-lowering treatments such as proprotein convertase subtilisin/kexin type 9 inhibitors may be considered as a good alternative [11]. Our patient was discharged to an acute rehabilitation facility with a tracheostomy tube and a PEG tube for support. He continues his recovery as of now, seven months following the diagnosis of IMNM. His PEG tube was removed in the seventh month, and his muscle weakness continues to gradually improve with physical therapy.

## Conclusions

Clinicians should have awareness about the wide spectrum of myopathies associated with statins. While IMNM is a rare condition, it is indeed devastating to the patient. Early recognition and prompt management with expert guidance are of paramount importance for better outcomes. Multidisciplinary approach at management with the help of cardiology, rheumatology, and internal medicine along with ancillary services, including physical therapy and occupational therapy, is of utmost importance. 
